# Placenta Accreta following Hysteroscopic Lysis of Adhesions Caused by Asherman's Syndrome: A Case Report and Literature Review

**DOI:** 10.1155/2018/6968382

**Published:** 2018-05-31

**Authors:** Yuko Sonan, Shigeru Aoki, Kimiko Enomoto, Kazuo Seki, Etsuko Miyagi

**Affiliations:** ^1^Perinatal Maternity and Neonatal Center of Yokohama City University Medical Center, Yokohama, Japan; ^2^Department of Obstetrics and Gynecology, Yokohama City University Hospital, Yokohama, Japan

## Abstract

Asherman's syndrome is defined as partial or complete obstruction of the uterine cavity primarily caused by intrauterine procedures and infections. Hysteroscopic adhesiolysis is commonly used to treat Asherman's syndrome. Although the frequency of placenta accreta is known to increase with pregnancy after hysteroscopic adhesiolysis, precise data remain unknown. We report a case of placenta accreta following hysteroscopic lysis of adhesions caused by Asherman's syndrome and IVF treatment and review the literature on placenta accreta following hysteroscopic adhesiolysis. It is necessary to consider placenta accreta as a complication of pregnancies after hysteroscopic adhesiolysis for Asherman's syndrome, particularly in those conceived using IVF.

## 1. Introduction

Asherman's syndrome is defined as partial or complete obstruction of the uterine cavity due to damage to the basal layer of the endometrium [1, 2] and is primarily caused by intrauterine procedures and infections often associated with miscarriage or curettage for postpartum placental retention [1].

Hysteroscopic adhesiolysis is commonly used to treat Asherman's syndrome. Although the frequency of placenta accreta is known to increase with pregnancy after hysteroscopic adhesiolysis, precise data remain unknown.

Here, we report a case of placenta accreta following hysteroscopic lysis of adhesions caused by Asherman's syndrome and IVF treatment and review the literature on placenta accreta following hysteroscopic adhesiolysis.

## 2. Case Presentation

The patient was a 40-year-old primiparous woman. She was diagnosed with submucosal fibroids by her previous gynecologist 5 years prior, based on chief complaints of atypical genital bleeding and hypermenorrhea. She underwent hysteroscopic myomectomy for one 1 cm sized and one 3 cm sized submucosal fibroid located between 2 and 3 o'clock in the uterine fundus. Asherman's syndrome was suspected after the patient exhibited secondary hypomenorrhea 10 months after surgery. Therefore, hysterosalpingography and magnetic resonance imaging (MRI) were performed. Intrauterine adhesions were suspected based on hysterosalpingography findings, while uterine cavity narrowing was identified using MRI. Hysteroscopy revealed filmy adhesions consistent with myomatous tissue at the excision site, and the patient was diagnosed with Asherman's syndrome.

Eight months after diagnosis, the patient underwent hysteroscopic adhesiolysis. The filmy adhesions observed on the left side of the fundus were easily separated with Hegar cervical dilators, and an intrauterine device was inserted after dilation. The patient was diagnosed with stage I Asherman's syndrome defined by European Society for Hysteroscopy classification of intrauterine adhesions, and menstrual flow returned to normal after the operation.

While the patient had a strong desire to bear children, her inability to conceive for 7 years led her to pursue in vitro fertilization (IVF). After having a miscarriage at 7 weeks of gestation, she underwent cervical dilatation and uterine curettage.

**Figure 1:**
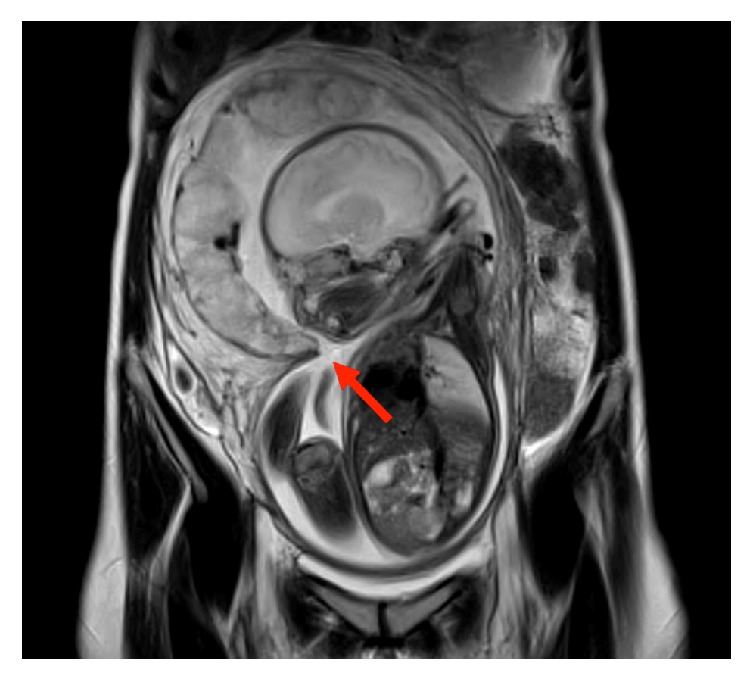
Semicircular stenosis, thought to influence adhesions in the uterus, was confirmed, and amniotic membrane sheets (tissues with a free edge visualized within the amniotic cavity) were identified (arrow).

Six months after the miscarriage, the patient became pregnant again through IVF and was referred to our hospital at 7 weeks of gestation. At 19 weeks of gestation, tissues with a free edge were visualized within the amniotic cavity using obstetric ultrasound and were determined to be amniotic sheets on MRI at 31 weeks of gestation (Figure 1). The course of pregnancy was uneventful thereafter, and an elective cesarean section was performed at 38 weeks and 2 days of gestation because of a breech presentation.

**Figure 2:**
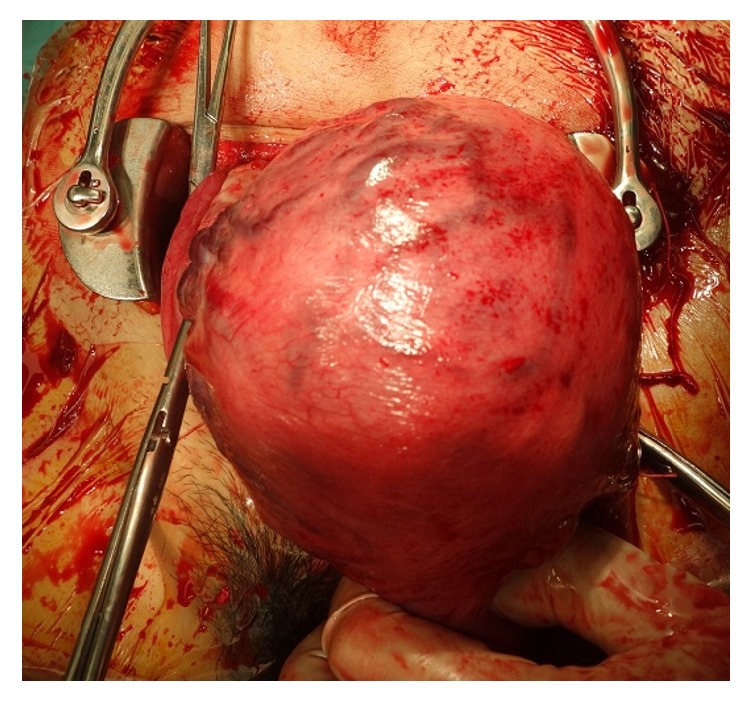
Intraoperative finding: blood vessels on the uterine surface at the placental implantation site are engorged.

**Figure 3:**
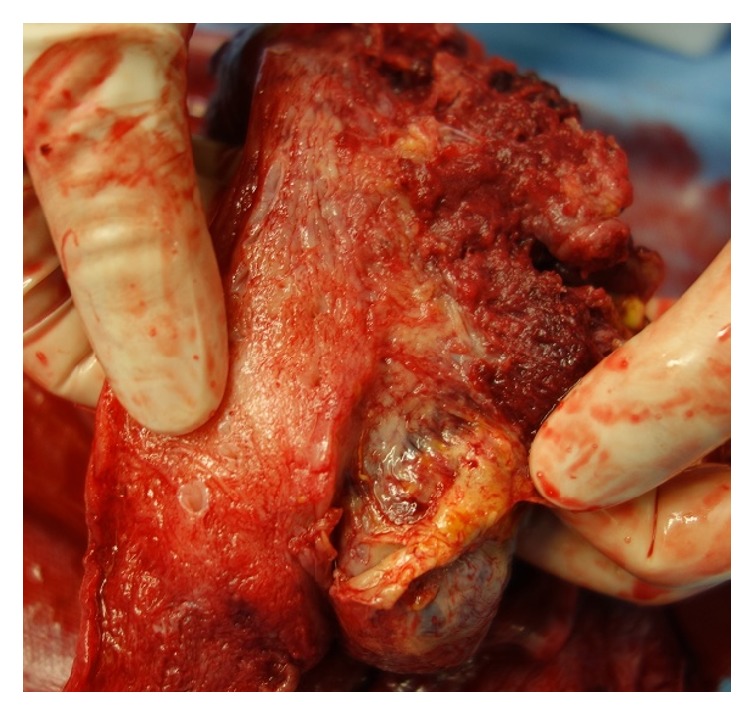
Resected uterus: placenta is present from the fundus to the posterior uterine wall. The placenta adheres diffusely to the myometrium, and partial thinning is visible at the fundus.

The placenta adhered to the uterine wall after childbirth and could not be easily separated manually. The blood vessels on the uterine surface at the placental implantation site were engorged (Figure 2), leading us to diagnose the patient with placenta increta. The placenta remained firmly adherent to the uterine wall, and although there was almost no bleeding from the uterine cavity, cesarean hysterectomy was performed after informed consent was obtained from the patient. In the abdominal cavity, 4 cm subserosal uterine fibroids were observed on the left side of the fundus, and adhesions thought to be caused by endometriosis were found in the right adnexa, posterior uterus, and anterior rectum. The operative time was 101 minutes, while the total blood loss was 1,584 ml (including amniotic fluid). Blood transfusion was not required. Macroscopic examination of the uterus after extraction showed the presence of placenta from the fundus to the posterior wall, diffusely adherent to the myometrium (Figure 3), along with partial thinning of the fundus.

Placenta increta was confirmed based on postpartum histological findings of placental villi invading the myometrium, without an interposed decidual plate.

The postoperative course was uneventful, and the patient was discharged in good health on the 7th postpartum day.

## 3. Discussion

This case highlights two points: placenta accreta must be considered as a potential complication in pregnancies after hysteroscopic adhesiolysis for Asherman's syndrome, and IVF may further increase the risk of placenta accreta.

First, we should be aware of the possibility of placental implantation disorders, placenta accrete in particular in pregnancies following hysteroscopic adhesiolysis. This patient underwent hysteroscopic adhesiolysis for intrauterine adhesions. Although approximately 90% of intrauterine adhesions are associated with intrauterine curettage in pregnant women, they can also occur in a nongravid uterus as a result of procedures like myomectomy and curettage that damage the endometrium [13]. Intrauterine adhesions are among the main long-term complications associated with hysteroscopic myomectomy [14], and Al-Inany [13] reported that multiple myomectomies are more likely to cause intrauterine adhesions than a single myomectomy. The amniotic sheets observed in this case may have been related to the intrauterine adhesions resulting from hysteroscopic myomectomy and the D&C performed because of a miscarriage.

Table 1 shows the results of a literature review performed by searching MEDLINE for pregnancy outcomes following hysteroscopic adhesiolysis for Asherman's syndrome [1–12]. Although the reported frequency of subsequent placenta accreta varies, a review of 12 studies published between 1986 and 2016 showed that 647 of such pregnancies ended in live birth. Of these, 17 women were diagnosed with placenta accreta, at a rate of 2.6% (17/647). Additionally, many, but not all, cases described adherent placenta and retained placenta without concomitant placenta accreta. Xiao et al. [15] reported that 64/201 cases (31.8%) had retained placenta, adherent placenta, or placenta accreta, and postpartum hemorrhage (PPH) occurred in 127/221 (63.2%) cases, suggesting that not only placenta accreta but also PPH must be considered in pregnancies after hysteroscopic adhesiolysis.

Second, IVF may increase the risk of placenta accreta.

The incidence of placenta accreta is significantly higher in IVF pregnancies than in spontaneous pregnancies [16]. Esh-Broder et al. [16] reported that the rate of placenta accreta in spontaneous pregnancies was 12/752 (1.2/1000), while that in IVF pregnancies was 30/24,441 (16/1,000). The higher rate of placenta accreta in IVF pregnancies may be due to the change in endometrial environment and morphological and structural changes to the endometrium due to the IVF treatment protocol (stimulation protocol) [16].

Intrauterine adhesions (IUA) severity is associated with greater reduction in fertility [1, 3, 17], thereby increasing the likelihood that affected patients will need to undergo IVF to have a successful pregnancy. The extent of endometrial loss increases with IUA severity, which likely confers a greater risk of placenta accreta. For this reason, doctors should be aware that IVF pregnancy following hysteroscopic adhesiolysis involves a greater risk of placenta accreta.

## 4. Conclusion

Placenta accreta should be considered as a complication of pregnancies after hysteroscopic adhesiolysis for Asherman's syndrome, particularly in those conceived using IVF.

## Figures and Tables

**Table 1:** Pregnancies after hysteroscopic adhesiolysis.

Study	Delivery following hysteroscopic adhesiolysis, *n*	Adherent placenta, *n* (%)	Placenta accreta, *n* (%)	Postpartum hemorrhage, *n* (%)
Chen et al. (2017) [1]	140	6 (4.3)	3 (2.1)	11 (7.9)
Fernandez et al. (2006) [2]	21	0	3 (14.3)	3 (14.3)
Roy et al. (2010) [3]	32	3 (9.4)	1 (3.1)	5 (12.5)
Yu et al. (2008) [4]	25	3 (12.0)	2 (8.0)	-
Zikopoulos et al. (2004) [5]	20	0	2 (10.0)	-
Capella-Allouc et al. (1999) [6]	9	0	2 (22.2)	-
Bhandari et al. (2015) [7]	10	1 (10.0)	0	1 (10.0)
Liu et al. (2014) [8]	49	1 (2.0)	2 (4.1)	2 (4.1)
Katz et al. (1996) [9]	59	0	0	0
Friedman et al. (1986) [10]	23	1 (8.7)	1 (8.7)	-
Valle and Sciarra (1988) [11]	114	-	1 (0.88)	-
Feng et al. (1999) [12]	145	1 (0.7)	0	-
Total	647	16 (2.5)	17 (2.6)	22 (3.4)
